# Biotechnological route for obtaining methyl esters from crambe oil (*Crambe abyssinica*)

**DOI:** 10.1186/1753-6561-8-S4-P215

**Published:** 2014-10-01

**Authors:** Monna Lisa Queiroz, Bruna Onorevoli, Gabriela Fontes, Laiza Krause, Heiddy Alvarez, Cláudio Dariva, Elina Caramão, Alini Fricks

**Affiliations:** 1 University Tiradentes, Institute for Research and Technology-ITP. Masters and Doctoral Program in Industrial Biotechnology / UNIT, Aracaju, SE, Brazil; 2 Institute of Chemistry, Federal University of Rio Grande do Sul, Porto Alegre, RS, Brazil; 3 Department of Exact Sciences, State University of Feira de Santana, Campus UEFS, Feira de Santana, Bahia, Brazil

## Introduction

The fatty acid esters synthesis by transesterification of oils to produce biodiesel commonly involves methanol or ethanol as acyl acceptor. The transesterification of vegetable oils catalyzed by lipases is an alternative process for obtaining biodiesel. These biocatalysts working under mild conditions of temperature, allow for easy recovery of glycerol, synthesis of specific alkyl esters and transesterification of triglycerides with high concentrations of free fatty acids. The seeds of crambe (*Crambe abyssinica*) have a high oil content and great potential for biodiesel production [1]. In this way, the present work aims at crambe oil transesterification with methanol catalyzed by immobilized lipase Novozyme 435 (*Candida antarctica*).

## Experimental

Crambe seeds were powdered and lyophilized for extraction of crude oil by pressing for 72h. For transesterification first was evaluated the effect of molar ratio (MR) methanol:oil in the range of 3:1 to 12:1 with reaction time of 6h and 3.36% (w/w) of enzyme. In the best MR evaluated the effect of reaction time (0.375-24h). The reactions were conducted in thermostated bath in reactor containing oil (1.5g-1.62 mmols), methanol, immobilized lipase and tert-butanol at 20% (w/w) with respect to the mass of oil. The quantification of fatty acid methyl esters (FAME) formed was performed by gas chromatography according to European standard EN14103, with minor modifications. The conversion was calculated based on a standard curve constructed with biodiesel 100% purity.

## Results and discussion

Factors such as presence and the molar ratio of organic solvent affecting the transesterification of vegetable oils. The lipase *C. antarctica *tolerates reaction media containing organic solvents, in particular hydrophobic as hexane, [2] but in transesterification reactions, the substrates used (alcohol) and reaction product (glycerol) this type are immiscible solvent, which might result adsorption of these polar molecules on the hydrophilic support of the immobilized enzyme and low reaction rates. To solve this problem tert-butanol at a concentration of 20% was used as co-solvent because it is moderately hydrophilic (log *P *= 0.80) being able to solubilize oil, methanol and glycerol formed by eliminating the negative effects of methanol and glycerol on enzyme activity [3]. The high values of MR negatively affect the reaction rate by cause enzyme denaturation see the large concentration of highly hydrophilic compound (methanol) in the reaction medium [4]. At low conversions were obtained with the FAME with MR 9:1 to 12:1 (24 and 17%), while conversions of 64 and 54% were obtained in the MR 3:1 and 6:1, respectively. The profile of the kinetics of formation of FAME showed reactional system in conditions of initial velocity reaction with 3h. From 12h the reaction system tends to equilibrium, reaching maximum conversion of 82% after 24h of reaction. The results show promising potential to obtain biodiesel from crude crambe by enzymatic route.
